# Proximity to Industrial Food Animal Production and Asthma Exacerbations in Pennsylvania, 2005–2012

**DOI:** 10.3390/ijerph14040362

**Published:** 2017-03-31

**Authors:** Sara G. Rasmussen, Joan A. Casey, Karen Bandeen-Roche, Brian S. Schwartz

**Affiliations:** 1 Department of Environmental Health and Engineering, Johns Hopkins Bloomberg School of Public Health, Baltimore, MD 21205, USA; srasmus7@jhu.edu; 2 Department of Environmental Science, Policy & Management, University of California, Berkeley, Berkeley, CA 94720, USA; joanacasey@berkeley.edu; 3 Department of Biostatistics, Johns Hopkins Bloomberg School of Public Health, Baltimore, MD 21205, USA; kbandee1@jhu.edu; 4 Department of Epidemiology and Health Services Research, Geisinger Health System, Danville, PA 17822, USA; 5 Department of Medicine, Johns Hopkins School of Medicine, Baltimore, MD 21205, USA

**Keywords:** asthma, asthma exacerbation, concentrated animal feeding operation (CAFO)

## Abstract

The research on industrial food animal production (IFAP) and asthma exacerbations in the United States has relied on small sample sizes and/or self-reported outcomes. We assessed associations of proximity to large-scale and densely stocked swine and dairy/veal IFAP with three types of asthma exacerbations: hospitalizations, emergency encounters, and oral corticosteroid (OCS) medication orders from Geisinger Clinic in Pennsylvania. We used a diagnosis code (*International Classification of Diseases, 9th Revision, Clinical Modification* code 493.x) and medication orders from electronic health records to identify these exacerbations among asthma patients (*n* = 35,269) from 2005–2012. We compared residential proximity to swine or dairy/veal IFAP (dichotomized as <3 miles (4.8 km) or ≥3 miles) among asthma patients with and without exacerbations and estimated odds ratios using multilevel logistic regression. In adjusted models, proximity to IFAP was associated (odds ratio (95% confidence interval)) with OCS orders (1.11 (1.04–1.19)) and hospitalizations (1.29 (1.15–1.46)), but not emergency encounters (1.12 (0.91–1.37)). This study contributes to growing evidence that IFAP may impact health, in this case clinically-documented asthma exacerbations. No prior study has evaluated the association of IFAP and clinically-documented asthma exacerbations in the United States.

## 1. Introduction

Living or attending school near industrial food animal production (IFAP)—large-scale, densely stocked, and highly specialized farms—has been linked to adverse health outcomes [1]. Of particular concern are asthma exacerbations. Asthma is a chronic condition characterized by recurring symptoms (including cough, wheezing, shortness of breath, and chest tightness) that affects over 25 million people in the United States [2,3]. IFAP facilities are a source of odors and several air pollutants, including particulate matter, hydrogen sulfide, and ammonia [4], and these air pollutants and odors have been associated with asthma exacerbations [2,5,6]. Most, but not all, studies on IFAP and asthma exacerbations have found IFAP to be a risk factor for decreased lung function and asthma symptoms [1,5,7,8,9,10,11,12,13,14,15]. However, the research on IFAP and asthma exacerbations in the United States has relied on small sample sizes and/or self-reported outcomes [5,7,8,9,10], and studies that have used clinically-documented events, from the Netherlands, reported no association or a protective association of IFAP and asthma exacerbations [14,15,16]. In this study, we used electronic health record (EHR) data from the Geisinger Clinic, located in central and northeastern Pennsylvania, to conduct a case-control study of the association between proximity to swine or dairy/veal IFAP and clinically-documents asthma exacerbations in a population of patients with asthma.

## 2. Materials and Methods

### 2.1. IFAP Data and Study Population

Swine and dairy/veal IFAP were identified in nutrient management plans from the Pennsylvania Department of Environmental Protection and County Conservation Districts in the Geisinger Clinic’s service area and adjacent counties, as described in a prior study [4]. In Pennsylvania, the Department of Environmental Protection and County Conservation Districts regulate IFAP, both concentrated animal feeding operations (CAFOs, defined as operations with >1000 animal equivalent units (AEUs; 1 AEU = 1000 lbs or 454 kg)) and concentrated animal operations (CAOs, defined as >2 AEUs/acre) under Act 38. Act 38 requires operators to produce nutrient management plans every two years. We located swine and dairy/veal IFAP in the Geisinger Clinic’s service area and adjacent counties using these plans and assigned each a latitude and longitude based on the location of operation’s center [4]. 

**Figure 1:**
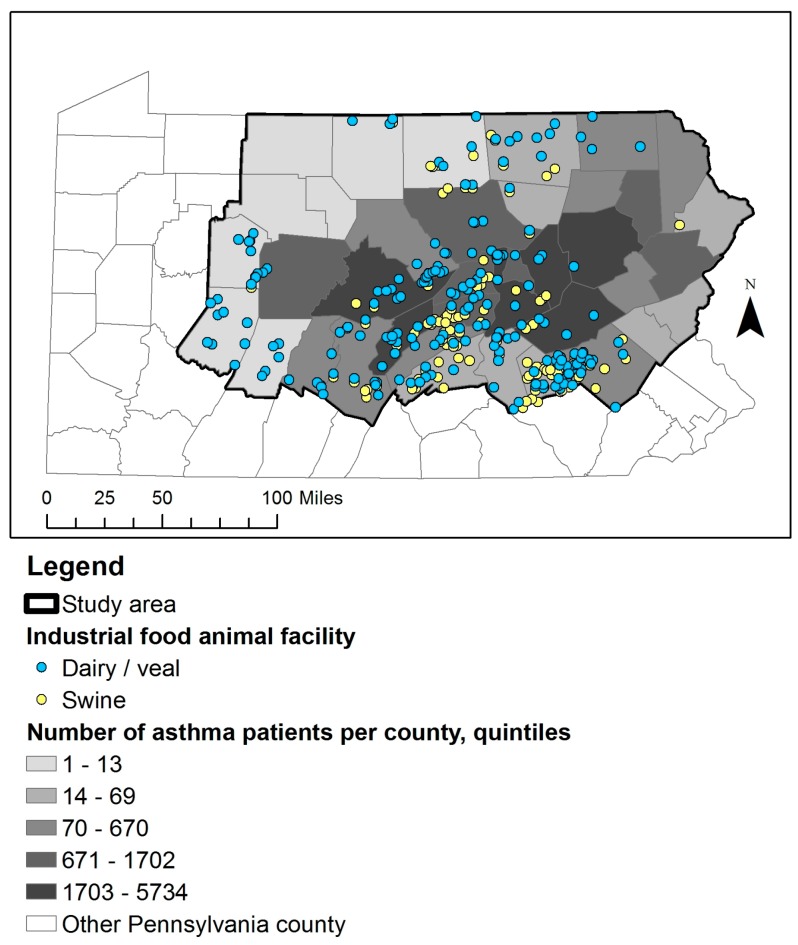
The location of swine and dairy/veal industrial food animal production facilities and the residential location of Geisinger Clinic asthma patients.

The study population consisted of primary patients with asthma, and we identified the study population from the Geisinger Clinic population, which is representative of the general population in the region, using methods described in a prior study [17]. Briefly, we considered patients with at least two encounters with asthma diagnoses on different days (using *International Classification of Diseases, 9th Revision, Clinical Modification* code 493.x), or at least one encounter with an asthma diagnosis and at least one medication order with an asthma diagnosis on different days, to be patients with asthma [18]. Patients were geocoded to their home addresses using ArcGIS Advanced for Desktop, v10.1 (Esri, Redlands, CA, USA) and restricted the study population to the Geisinger Clinic’s service area and adjacent counties (Figure 1). We identified the following asthma exacerbations among the study population in the EHR: hospitalizations (2005–2012), emergency encounters (2005–2012), and new oral corticosteroid (OCS) orders (2008–2012; OCS orders were only consistently captured in the EHR from 2008 and on). We used both primary and secondary diagnosis codes for asthma for hospitalizations and emergency encounters because we were unable to differentiate between asthma exacerbations that led to hospitalizations and asthma exacerbations that occurred while patients were hospitalized. We identified new OCS medication orders for asthma exacerbations by excluding standing orders and requiring that the outpatient visit reason or medication order diagnosis was asthma-related [17]. The study was approved by the Geisinger Institutional Review Board (approval number 2013-0114, with an Institutional Review Board authorization agreement with Johns Hopkins Bloomberg School of Public Health).

### 2.2. Comparison Subjects and Matching

For each type of asthma exacerbation, we compared asthma patients with at least one exacerbation of that type during the study period to comparison subjects, who were asthma patients with no or less severe exacerbations (OCS order < emergency encounter < hospitalization) during the study period. We frequency-matched comparison subjects to cases on age category (5–12, 13–18, 19–44, 45–61, 62–74, 75 + years), sex (male, female), and year of event. For cases, the date of their first event was used as the year for frequency-matching and as the date to assign time-varying covariates. For comparison subjects, we needed a date for frequency matching by year and to assign time-varying covariates, so we used randomly selected a date on which the patient had contact with the health system. Hospitalization cases were matched to comparison subjects at 1:3, emergency department cases were matched at 1:5, and OCS cases were matched at 1:1. 

### 2.3. Statistical Analysis

For each outcome, we evaluated unadjusted and adjusted multilevel logistic regression models with a random intercept for community to account for patient clustering within communities (in cities, census tracts; in townships and boroughs, minor civil divisions). Proximity to IFAP was defined as a swine or dairy/veal operation within a three mile (4.8 km) buffer of the patient’s residence; this distance was chosen based on a prior study [9]. Covariates in the adjusted model included those created using the EHR: age category, year, sex, self-reported race/ethnicity (white, black, Hispanic, other/missing; race/ethnicity is a well-documented confounder in asthma studies [2]), season, smoking status, family history of asthma, overweight/obesity (using body mass index (BMI) percentile for children and BMI (kg/m^3^) for adults [19]), Medical Assistance (a means tested program that is an indicator of low family socioeconomic status), and type 2 diabetes; and those created using patients’ geocoded coordinates: distance to nearest major and minor road and community socioeconomic deprivation (CSD) [4,17,20,21]. Because distance from patient address to the closer of the two major Geisinger hospitals, where 96% of asthma hospitalizations and 85% of asthma emergency encounters took place, was a potential confounding variable, it was included in the hospitalization and emergency encounter models. Distance to hospital was not included in the OCS model, as OCS orders were handled at outpatient locations and over the phone. Continuous covariates were included with linear and quadratic terms to allow for non-linearity.

### 2.4. Sensitivity Analyses

We conducted several sensitivity analyses. Because we were interested in effect modification by age, we evaluated whether associations between IFAP and asthma exacerbations were different among children and adults by adding a cross-product of proximity to IFAP and an indicator for childhood age (5–18 years) to the final model for each outcome. To evaluate the impact of including patients in urban areas, we completed a sensitivity analysis that excluded patients in cities. We evaluated associations among patients who had more than one exacerbation of a given type by repeating the primary analysis only including cases with more than one of a given type of exacerbation. Because of concern about confounding by distance to hospital, in a sensitivity analysis for the hospitalization and emergency department outcomes, we individually matched (1:1) cases to comparison subjects on age, sex, and distance to hospital in five mile increments. Because of the individual matching, for this analysis we evaluated unadjusted and adjusted conditional logistic regressions, which included the same covariates as in the primary analysis. To evaluate the impact of using patients with asthma exacerbations less severe than the cases as comparison subjects on our associations, or each outcome, we completed a sensitivity analysis in which asthma patients with that type of exacerbation were compared to patients with no asthma exacerbations of any type during the study period, and we then evaluated associations using crude and adjusted logistic regressions. 

## 3. Results

### 3.1. Description of Study Population

We identified 35,269 patients with asthma in the study area. The study population was 92% white, 58% female, had a median age of 31 years in 2012, and 59, 30, and 11% of the population lived in townships, boroughs, and cities, respectively. The hospitalization analysis included 3552 cases and 10,640 comparison subjects, the emergency encounter analysis included 1445 cases and 7225 comparison subjects, and the OCS analysis included 13,137 cases and 13,044 comparison subjects. Cases for each of the three outcomes were more likely than comparison subjects to have a family history of asthma, be current smokers, have Medical Assistance, be obese, and live in communities in the highest quartile of CSD (Table 1 and Table 2). Cases in the hospitalization and emergency analyses, but not the OCS analysis, were less likely to be white non-Hispanic and more likely to live closer to major and minor roads. We identified 123 swine and 203 dairy/veal operations, and 8399 (24%) patients lived within 3 miles of one of these (Figure 1).

### 3.2. Association of IFAP and Asthma Exacerbations

In unadjusted models, proximity to IFAP was not associated with hospitalizations (odds ratio OR = 1.11, 95% CI: 0.97–1.27) or emergency encounters (OR = 0.90, 95% CI: 0.70–1.16), but was associated with increased odds of OCS orders (OR = 1.11, 95% CI: 1.03–1.19). After distance to hospital was added to the model, proximity to IFAP was associated with increased odds in the hospitalization model and was not associated in the emergency encounters model. Adding the remaining covariates did not substantially change the associations (Table 3). 

### 3.3. Results of Sensitivity Analyses

The *p*-value of the cross-product of proximity to IFAP and an indicator for childhood age were 0.53, 0.73, and 0.82 for the hospitalization, emergency department, and OCS analyses, respectively, indicating that associations in children and in adults were similar. The odds ratios from the sensitivity analysis that excluded patients in cities (for hospitalizations, OR = 1.29, 95% CI: 1.14–1.46; for emergency encounters, OR = 1.09, 95% CI: 0.89–1.34; and for OCS orders, OR = 1.12, 95% CI: 1.04–1.20) were similar to those in the primary analysis. In the sensitivity analysis that included cases with more than one of a given type of event, we found similar results as in the primary analysis (for hospitalizations, OR = 1.39, 95% CI: 1.09–1.76; for emergency encounters, OR = 1.12, 95% CI: 0.91–1.37; and for OCS orders, OR = 1.14, 95% CI: 1.02–1.27). In the sensitivity analysis that individually matched cases to comparison subjects on age, sex, and distance to hospital, in adjusted models, associations were similar to those from the primary analysis for hospitalizations (OR = 1.24, 95% CI: 1.09–1.40) and for emergency encounters (OR = 1.07, 95% CI: 0.86–1.33). In the sensitivity analysis that used patients with no asthma exacerbations during the study period as comparison subjects, associations were similar to the primary analysis for hospitalizations (OR = 1.29, 95% CI: 1.13–1.46) and stronger though still not statistically significant for emergency encounters (OR = 1.19, 95% CI: 0.97–1.46). 

## 4. Discussion

We evaluated associations of IFAP with three kinds of clinically-documented asthma exacerbations, and conducted a number of sensitivity analyses, in a large population of asthma patients in a region with many IFAP facilities. We found 11% and 29% increased odds of OCS orders and asthma hospitalizations, respectively, among asthma patients living within 3 miles of IFAP, compared to living farther away. We did not observe effect modification by childhood age. We found similar results using individual matching on distance to hospital, and using patients with no asthma exacerbations during the study period as the comparison group. 

Our study found that distance to hospital was an important confounder in the hospitalization analysis. A study from the Netherlands found that proximity to livestock farms (<500 m from home address) was negatively associated with the number of patient contacts with general practitioners (including encounters, home visits, and telephone consultations), but in contrast to our study, their association was unchanged when distance to general practice was added to the model [16]. 

We report different results than two prior studies from the Netherlands that used clinically-documented asthma (as opposed to self-reported) to assess the association of IFAP and asthma. The first study evaluated asthma prevalence, not exacerbations, so it is not relevant to our findings [14]. The other study concluded that proximity to IFAP was not associated with asthma exacerbations [15]. They found no association of distance to nearest swine or poultry farm with asthma exacerbations, as measured by an increase in the dose or a new medication order for asthma medications. In contrast to that study, we found an increased risk of new OCS orders for asthma and for asthma hospitalizations among asthma patients living close to swine or dairy/veal IFAP compared to farther away.

Our study had limitations. The buffer proximity approach treated swine and dairy/veal facilities as the same, though emissions may differ between the two, and did not incorporate animal count, IFAP building characteristics, or wind direction. Furthermore, we did not have information on poultry IFAP and we did not take air samples. Proximity was based on distance from residential address (ascertained in 2013) to closest IFAP operation, so it might misclassify exposures at school or work, or if the patient had recently moved, though an analysis in a prior study of this population indication low residential mobility [8]. We did not have information on study participants’ housing conditions, which could contribute to asthma exacerbations. We did not ascertain events that occurred outside the Geisinger system. The EHR only captures events for which patients seek care, so we do not have data on asthma symptoms that patients could treat on their own without medical care. We only collected information on IFAP in 2010 and 2011, and we assumed facilities were in the same location for the entire study period. However, we verified the locations of a random sample of 20 IFAP in Google Earth’s historical aerial imagery, and all were present in the images since at least 2005, so we believe this was a reasonable assumption. Strengths of this study included a large sample size consisting of both children and adults. We incorporated both swine and dairy/veal IFAP, unlike previous studies in the United States that only incorporated swine IFAP [1,8,9]. We used asthma exacerbations documented in an EHR instead of relying on self-reported outcomes [1,5,8], which can result in recall bias [22].

## 5. Conclusions

Our study observed an association of clinically-documented asthma exacerbations, a serious public health concern, in relation to IFAP in Pennsylvania. The finding is biologically plausible and was robust in a number of relevant sensitivity analyses.

## Figures and Tables

**Table 1:** Descriptive statistics of cases and comparison subject characteristics by exacerbation type.

Variable, *n* (% ^a^), Unless Specified	Hospitalization		Emergency Visit		OCS ^b^	
	Case *n* = 3552	Comparison Subject *n* = 10,640	Case *n* = 1445	Comparison Subject *n* = 7225	Case *n* = 13,137	Comparison Subject *n* = 13,044
Race/ethnicity						
White non-Hispanic	3297 (92.8)	10,065 (94.6)	1218 (84.3)	6720 (93)	12,126 (92.3)	11,998 (92)
Black non-Hispanic	107 (3)	257 (2.4)	124 (8.6)	221 (3.1)	428 (3.3)	468 (3.6)
Hispanic/other/missing	148 (4.1)	318 (3.0)	103 (7.1)	284 (3.9)	583 (4.5)	578 (4.5)
	*p* ^c^ < 0.01		*p* < 0.01		*p* = 0.43	
Family history of asthma	404 (11.4)	999 (9.4)	265 (18.3)	871 (12.1)	1668 (12.7)	1354 (10.4)
	*p* < 0.01		*p* < 0.01		*p* < 0.01	
Smoking status						
Never	1529 (43)	5567 (52.3)	656 (45.4)	4148 (57.4)	7424 (56.5)	7975 (61.1)
Current	895 (25.2)	1913 (18)	276 (19.1)	1100 (15.2)	2219 (16.9)	1777 (13.6)
Former/missing	1128 (31.8)	3160 (29.7)	513 (35.5)	1977 (27.4)	3494 (26.6)	3292 (25.2)
	*p* < 0.01		*p* < 0.01		*p* < 0.01	
Medical Assistance	1094 (30.8)	2024 (19)	542 (37.5)	1991 (27.6)	3683 (28.0)	3369 (25.8)
	*p* < 0.01		*p* < 0.01		*p* < 0.01	
Body mass index						
Normal/underweight	794 (22.4)	2873 (27)	445 (30.8)	2574 (35.6)	3887 (29.6)	4693 (36)
Overweight	786 (22.1)	2758 (25.9)	304 (21)	1708 (23.6)	3103 (23.6)	3164 (24.3)
Obese	1972 (55.5)	5009 (47.1)	696 (48.2)	2943 (40.7)	6147 (46.8)	5187 (39.8)
	*p* < 0.01		*p* < 0.01		*p* < 0.01	
Type 2 diabetes	558 (15.7)	1033 (9.7)	114 (7.9)	405 (5.6)	1051 (8.0)	1010 (7.7)
	*p* < 0.01		*p* < 0.01		*p* = 0.44	
Age ^d^ (years)						
5–12	301 (8.5)	903 (8.5)	376 (26)	1880 (26)	2975 (22.6)	2975 (22.8)
13–18	222 (6.3)	666 (6.3)	156 (10.8)	780 (10.8)	1325 (10.1)	1298 (10)
19–44	1347 (37.9)	4041 (38)	576 (39.9)	2880 (39.9)	4065 (30.9)	4065 (31.2)
45–61	946 (26.6)	2838 (26.7)	234 (16.2)	1170 (16.2)	3177 (24.2)	3110 (23.8)
62–74	470 (13.2)	1410 (13.3)	70 (4.8)	350 (4.8)	1146 (8.7)	1146 (8.8)
75+	266 (7.5)	782 (7.3)	33 (2.3)	165 (2.3)	449 (3.4)	450 (3.4)
Female	2502 (70.4)	7506 (70.5)	868 (60.1)	4340 (60.1)	8133 (61.9)	8040 (61.6)
Year						
2005	527 (14.8)	1581 (14.9)	166 (11.5)	830 (11.5)		
2006	504 (14.2)	1512 (14.2)	147 (10.2)	735 (10.2)		
2007	436 (12.3)	1308 (12.3)	195 (13.5)	975 (13.5)		
2008	378 (10.6)	1134 (10.7)	179 (12.4)	895 (12.4)	3356 (25.5)	3356 (25.7)
2009	431 (12.1)	1293 (12.2)	206 (14.3)	1030 (14.3)	2986 (22.7)	2986 (22.9)
2010	398 (11.2)	1194 (11.2)	182 (12.6)	910 (12.6)	2405 (18.3)	2378 (18.2)
2011	422 (11.9)	1266 (11.9)	174 (12.0)	870 (12.0)	2342 (17.8)	2342 (18.0)
2012	456 (12.8)	1352 (12.7)	196 (13.6)	980 (13.6)	2048 (15.6)	1982 (15.2)

^a^ Percentages may not add to 100 due to rounding; ^b^ Oral corticosteroid (OCS) medication orders; ^c^ For categorical variables, the *p*-value reported is from a chi-squared test; ^d^ Cases were frequency-matched to comparison subjects on age, sex, and year.

**Table 2:** Place-level descriptive statistics of cases and comparison subjects by exacerbation type.

Variable, *n* (% ^a^), Unless Specified	Hospitalization		Emergency Visit		OCS ^b^	
	Case *n* = 3552	Comparison Subject *n* = 10,640	Case *n* = 1445	Comparison Subject *n* = 7225	Case *n* = 13,137	Comparison Subject *n* = 13,044
IFAP ^c^ within 3 miles of home address	832 (23.4)	2706 (25.4)	214 (14.8)	1849 (25.6)	3350 (25.5)	3042 (23.3)
	*p* ^d^ = 0.02		*p* < 0.01		*p* < 0.01	
Community socioeconomic deprivation, quartiles						
1	870 (24.5)	2839 (26.7)	289 (20)	1935 (26.8)	3424 (26.1)	3536 (27.1)
2	815 (22.9)	2573 (24.2)	298 (20.6)	1728 (23.9)	3093 (23.5)	3129 (24.0)
3	855 (24.1)	2694 (25.3)	345 (23.9)	1811 (25.1)	3389 (25.8)	3203 (24.6)
4	1012 (28.5)	2534 (23.8)	513 (35.5)	1751 (24.2)	3231 (24.6)	3176 (24.3)
	*p* < 0.01		*p* < 0.01		*p* = 0.06	
Distance to nearest major road, miles (mean)	1.59	1.66	1.35	1.73	1.69	1.69
	*p* = 0.16		*p* < 0.01		*p* = 0.91	
Distance to nearest minor road, miles (mean)	0.92	1.09	0.62	1.12	1.09	1.09
	*p* < 0.01		*p* < 0.01		*p* = 0.87	
Distance to hospital, miles (mean)	19.4	32.4	10.3	32.9	32.5	30.8
	*p* < 0.01		*p* < 0.01		*p* < 0.01	

^a^ Percentages may not add to 100 due to rounding; ^b^ Oral corticosteroid (OCS) medication orders; ^c^ Industrial food animal production; ^d^ For continuous variables, the *p*-value reported is from a *t*-test. For categorical variables, the *p*-value reported is from a chi-squared test.

**Table 3:** Associations of proximity to industrial food animal production (defined as a facility within 3 miles of home address) and asthma exacerbation outcomes.

Variable (95% CI ^a^)	Outcome		
	Asthma Hospitalizations ^b^	Asthma Emergency Department Visits ^b^	New Asthma OCS ^c^ Orders ^d^
Proximity to industrial food animal production ^e^	1.29 (1.15–1.46)	1.12 (0.91–1.37)	1.11 (1.04–1.19)
Race/ethnicity (ref: white non-Hispanic)			
Black non-Hispanic	1.05 (0.81–1.36)	1.86 (1.4–2.46)	0.84 (0.73–0.97)
Hispanic	1.23 (0.99–1.54)	1.47 (1.11–1.93)	0.95 (0.84–1.08)
Family history of asthma (ref: no)	1.27 (1.11–1.45)	1.68 (1.4–2.01)	1.27 (1.17–1.37)
Smoking status (ref: never)			
Current	1.45 (1.3–1.62)	1.35 (1.12–1.63)	1.39 (1.29–1.5)
Former	1.18 (1.08–1.3)	1.45 (1.25–1.68)	1.15 (1.08–1.22)
Medical Assistance (ref: no)	1.87 (1.69–2.07)	1.32 (1.13–1.54)	1.06 (0.998–1.13)
Body mass index (ref: normal/underweight)			
Overweight	1.07 (0.95–1.21)	1.07 (0.89–1.28)	1.24 (1.16–1.33)
Obese	1.36 (1.22–1.51)	1.3 (1.11–1.52)	1.53 (1.43–1.62)
Type 2 diabetes (ref: no)	1.63 (1.43–1.85)	1.29 (0.99–1.69)	0.93 (0.84–1.03)
Community socioeconomic deprivation (ref: quartile 1)			
Quartile 2	0.97 (0.83–1.13)	1.28 (1.01–1.63)	0.99 (0.91–1.08)
Quartile 3	0.97 (0.83–1.14)	1.41 (1.10–1.82)	1.01 (0.93–1.11)
Quartile 4	0.94 (0.79–1.12)	1.41 (1.10–1.82)	0.98 (0.89–1.08)
Distance to nearest major arterial road, z-transformed			
z-transformed	0.94 (0.84–1.04)	1.10 (0.93–1.31)	0.97 (0.91–1.03)
squared	1.07 (1.02–1.12)	1.04 (0.97–1.12)	1.02 (0.99–1.05)
Distance to nearest minor arterial road			
z-transformed	1.08 (0.97–1.19)	1.03 (0.87–1.22)	0.98 (0.92–1.04)
squared	0.998 (0.95–1.05)	0.89 (0.79–1.003)	1.003 (0.98–1.03)
Distance to nearest Geisinger hospital			
z-transformed	0.46 (0.42–0.51)	0.10 (0.08–0.13)	
squared	1.14 (1.07–1.21)	1.35 (1.09–1.66)	f

^a^ Confidence interval; ^b^ Multilevel models with a random intercept for community, adjusted for race/ethnicity (white, black, Hispanic, other), family history of asthma (yes, no), smoking status (never, former, current, missing), Medical Assistance (yes vs. no), overweight/obesity (normal, body mass index (BMI) < 85th percentile or BMI < 25 kg/m^2^; overweight, BMI = 85th < 95th percentile or BMI = 25 < 30 kg/m^2^; obese, BMI ≥ 95th percentile or BMI ≥ 30 kg/m^2^, for children and adults, respectively; BMI missing), type 2 diabetes (yes vs. no), community socioeconomic deprivation (quartiles), distance to nearest major and minor arterial road (truncated at the 98th percentile, meters, z-transformed), squared distance to nearest major and minor arterial road (truncated at the 98th percentile, meters, z-transformed), distance to nearest Geisinger hospital (truncated at the 98th percentile, kilometers, z-transformed), squared distance to nearest Geisinger hospital (truncated at the 98th percentile, kilometers, z-transformed), age category (5–12, 13–18, 19–44, 45–61, 62–74, 75+ years), sex (male, female), and year of event (2005, 2006, 2007, 2008, 2009, 2010, 2011, 2012); ^c^ Oral corticosteroid; ^d^ Multilevel model with a random intercept for community, adjusted for race/ethnicity (white, black, Hispanic, other), family history of asthma (yes, no), smoking status (never, former, current, missing), Medical Assistance (yes vs. no), overweight/obesity (normal, body mass index (BMI) < 85th percentile or BMI < 25 kg/m^2^; overweight, BMI = 85th < 95th percentile or BMI = 25 < 30 kg/m^2^; obese, BMI ≥ 95th percentile or BMI ≥ 30 kg/m^2^, for children and adults, respectively; BMI missing), type 2 diabetes (yes vs. no), community socioeconomic deprivation (quartiles), distance to nearest major and minor arterial road (truncated at the 98th percentile, meters, z-transformed), squared distance to nearest major and minor arterial road (truncated at the 98th percentile, meters, z-transformed), age category (5–12, 13–18, 19–44, 45–61, 62–74, 75+ years), sex (male, female), and year of event (2008, 2009, 2010, 2011, 2012); ^e^ Within 3 miles of home address; f, Distance to hospital was not included in this model.
